# Cardiac-homing peptide-targeted colchicine-based drugstores with inhibiting inflammatory response for myocardial infarction improvement

**DOI:** 10.1016/j.mtbio.2026.103212

**Published:** 2026-05-13

**Authors:** Xing Zhang, Wenhua Xu, Jie Song, Huanhuan Ding, Shujie Yu, Nan Yang, Dilare Taiwaikuli, Yemin Chen, Yanmei Lu, Baopeng Tang, Zhongxiong Fan, Xianhui Zhou

**Affiliations:** a Department of Cardiac Pacing and Electrophysiology, Xinjiang Key Laboratory of Cardiac Electrophysiology and Remodeling, The First Affiliated Hospital of Xinjiang Medical University, Urumqi, 830054, China; b School of Pharmaceutical Sciences, Institute of Materia Medica, Xinjiang University, Urumqi, 830017, China; c Xinjiang Key Laboratory of Biological Resources and Genetic Engineering, College of Life Science and Technology, Xinjiang University, Urumqi, 830017, China; d Department of Cardiology, Changji Prefecture People's Hospital in Xinjiang Uygur Autonomous Region, No.303 Yan'an Road, Changji City, Xinjiang, 831100, China; e School of Public Health, Xinjiang Medical University, China

**Keywords:** Colchicine, Liposomes, Myocardial infarction, Cardiac-homing peptide

## Abstract

One of the primary challenges in addressing cardiac injury is the limited cardiac specificity of therapeutic drugs. Lipid nanoparticles (LNPs) are a relatively novel class of delivery vehicles used for administering RNA and various drugs. A major challenge in the treatment of cardiac injury remains the poor cardiac specificity of most therapeutic agents. Despite their promise as a relatively novel delivery platform for RNA and other payloads, LNPs do not adequately overcome this limitation. Nevertheless, these LNPs exhibited preferential accumulation in the liver rather than the heart. To address this off-target biodistribution and enhance delivery efficiency to cardiac tissue, surface functionalization with cardiac-homing peptides (CHP) was performed. In this study, the CHP-functionalized LNP loaded with colchicine (termed as CCP) was developed as a targeted therapeutic platform for myocardial repair. This novel drug delivery system successfully localizes to the ischemic myocardium and precisely releases CCP to hypoxic, mildly acidic cardiomyocytes. In conclusion, CCP presents a promising platform for the sustained and localized delivery of drugs to the heart, thereby enhancing the therapeutic efficacy for cardiovascular diseases.

## Graphical abstract

**Figure:**
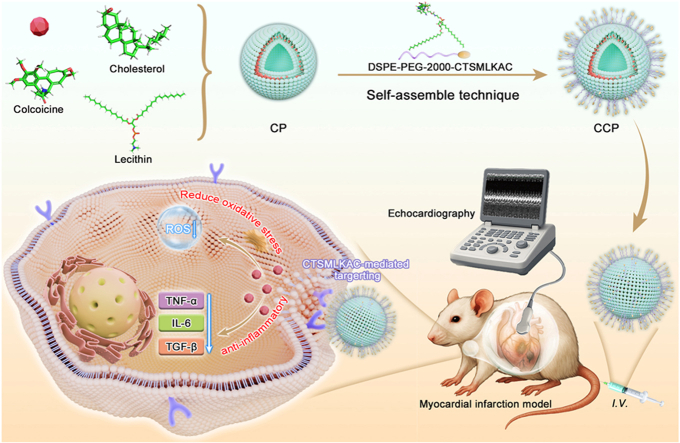

## 1 Introduction

Cardiovascular diseases, especially myocardial infarction (MI), are still among the leading causes of morbidity and mortality worldwide [1]. MI occurs due to the sudden blockage of coronary arteries, causing irreversible myocardial damage [2]. Current treatment strategies mainly aim to reduce infarct area and manage symptoms. Nevertheless, they are often insufficient in promoting long-term myocardial repair and functional recovery. Therefore, there is an urgent demand for new therapeutic approaches that can enhance cardiac regeneration and target the underlying pathological mechanisms of MI. Moreover, MI is known to trigger ventricular arrhythmias (VAs) and sudden cardiac death [3]. These are closely related to sympathetic neural remodeling, which is characterized by cardiac nerve sprouting and sympathetic hyperinnervation [4].

After MI, the heart undergoes a complex pathological remodeling process. This process involves notable increases in inflammatory response, overproduction of reactive oxygen species (ROS), and sympathetic neural remodeling [5,6].Inflammation promotes the generation of ROS, which enhances nerve injury and remodeling [7].ROS damage cell membranes, proteins, and DNA, triggering apoptosis of cardiomyocytes and tissue necrosis [8,9]. This cycle not only exacerbates myocardial injury but also indirectly promotes neural remodeling by continuously stimulating the production of NGF and the activation of neural-related pathways [10].This long-standing sympathetic neural remodeling is one of the core mechanisms leading to arrhythmia and sudden death [5,11,12].All in all, neural remodeling, inflammation, and ROS after MI are not isolated events. Instead, they form a dynamic, interdependent, and mutually amplifying pathological network [13,14].

Colchicine (COL) is a classic anti-inflammatory alkaloid mainly used for treatment of gout and pericarditis. It exerts its pharmacological effects by binding with high affinity to the *α/β*-tubulin heterodimer, which inhibits microtubule polymerization [15,16]. Notably, COL's anti-inflammatory effects are multimodal. Recent landmark trials (LoDoCo, LoDoCo2, COLCOT) have confirmed COL's cardioprotective efficacy in reducing major adverse cardiovascular events. However, the clinical utility of COL is limited by its narrow therapeutic index and dose-dependent adverse effects, particularly gastrointestinal toxicity and myelosuppression [17,18]. These limitations underscore the need for innovative drug delivery platforms to enhance safety profile while maintaining therapeutic efficacy [[18], [19], [20], [21], [22]].

**Scheme 1:**
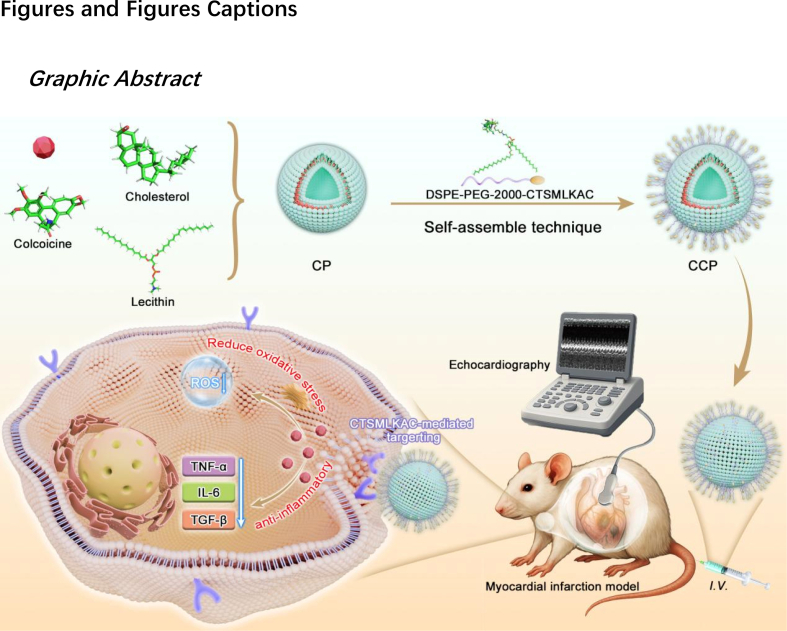
Scheme illustration of construction of cardiac-homing peptide-targeted colchicine-based CCP nanodrugs and their improvement effect by inhibiting inflammatory response.

Therefore, developing a nanoscale delivery system capable of overcoming bottlenecks—such as complex fabrication, poor cardiac targeting, and inefficient cellular uptake—is essential for safe and localized MI management. Inspired by the precision of targeted biomaterials, as depicted in Scheme 1, we designed a heart-specific delivery platform by integrating COL with CHP and clinical-grade phosphatidylcholine (PC). CCP was engineered to significantly enhance myocardial targeting efficiency. Leveraging the multifunctional properties of COL as a small-molecule agent [13], we reasoned that the integration of COL, CHP, and PC would not only endow the nanomedicine with superior cardiac homing capabilities but also simplify the structural complexity of the nanodrug. Herein, we report the fabrication of CCP via a robust self-assembly technique for synergistic MI therapy. Notably, once these nanodrugs penetrate the ischemic myocardium through peptide-mediated targeting, they achieve drug release, effectively ameliorating inflammation and maladaptive neural remodeling.

## 2 Results

### 2.1 Fabrication and characterization of CP

**Fig. 1:**
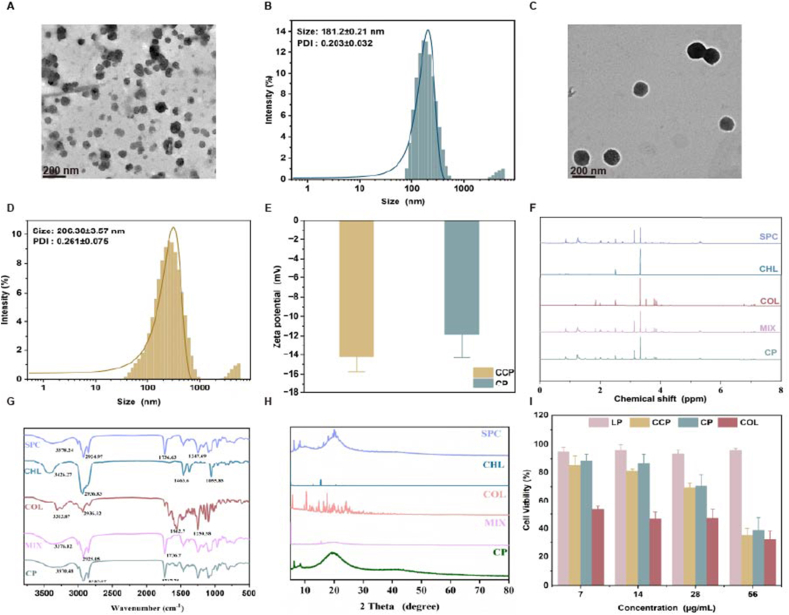
**Characterization of CP and CCP.** (A) the TEM image of CP. (B) the particle size distribution of CP. (C) the TEM image of CCP. (D) the particle size distribution of CCP. (E) Zeta potential analysis of CP and CCP. (F) the ^1^H NMR pattern of CP. (G) the FTIR of CP. (H) the XRD pattern of CP. (I) the effect of LP, CCP, CP, and COL on cell viability at different concentrations.

In this study, CP was successfully prepared using the thin-film hydration method. Transmission electron microscopy (TEM) images revealed that the CP exhibited a characteristic spherical morphology (Fig. 1A). The hydrodynamic diameter of CP was determined to be *ca.*181.2 nm, with a narrow polydispersity index (PDI) of 0.203 (Fig. 1B), suggesting a uniform size distribution. To enhance colloidal stability and cardiac-targeting efficiency, the CP were functionalized with a PEGylated cardiac-homing peptide (CSTSMLKAC). After surface functionalization, TEM observations revealed that the CCP retained their characteristic spherical morphology (Fig. 1C)**.** The hydrodynamic diameter of CCP was determined to be *ca*. 206 nm with a narrow polydispersity index (PDI) of 0.261 (Fig. 1D), suggesting a relatively uniform size distribution. The ζ potentials of CP and CCP were determined as *ca* -11.94 mV and 14.73 mV respectively (Fig. 1E), which was advantageous for their colloidal stability due to electrostatic repulsion. Notably, as depicted in the ^1^H NMR analysis, it was confirmed that soybean phosphatidylcholine (SPC) and cholesterol (CHL) integrated to form a stable lipid bilayer structure, a process primarily driven by hydrophobic interactions (Fig. 1F). Subsequently, to provide further insights into the molecular interactions and the chemical composition of the as-prepared system, FT-IR spectroscopy was performed (Fig. 1G). As displayed in the spectra, the physical mixture (MIX) exhibited an obviously additive effect of its individual components, with characteristic vibrational peaks corresponding to amino (N–H), alkyl chain (C–H), and ester (C=O) groups. Specifically, the peak at *ca.*3370 cm^−1^ was assigned to N–H stretching vibrations, confirming the presence of COL-associated amino groups and suggesting that COL was successfully encapsulated within the liposomal matrix. Furthermore, the characteristic peak at *ca.*2926 cm^−1^ was attributed to C–H stretching vibrations from the long-chain fatty acids of SPC or CHL, which constitute the hydrophobic bilayer membrane. The absorption at *ca.*1738 cm^−1^ corresponded to C=O stretching, further substantiating the structural integrity and stable formulation of the as-prepared CP. Next, to further reveal the crystalline state and distribution of individual components within the lipid matrix, X-ray diffractometry (XRD) was performed (Fig. 1H). As illustrated in the XRD patterns, both COL and SPC in their raw forms displayed characteristic crystalline peaks. In sharp contrast, the XRD pattern of the resulting CP exhibited significantly attenuated and broadened diffraction peaks in the 15° – 25° 2θ region, indicating a substantial suppression of SPC crystallinity. This phenomenon could be explained by the fact that the formulation of the liposomal bilayer inherently increases molecular disorder, while the incorporation of COL further disrupts the lipid packing order. These results substantiated that COL was no longer in a purely crystalline state but was homogeneously dispersed within the PC matrix. Taken together, these results substantiated that the structural stability of the CP system was driven by the synergistic interplay of hydrophobic interactions, hydrogen bonding, and the formation of covalent ester linkages. This confirmed that COL was chemically integrated within the liposomal architecture, rather than being merely physically encapsulated. Both the CP and the CCP dispersions appeared as white, translucent liquids. Notably, the CCP dispersion exhibited a more homogeneous state and a distinct Tyndall effect, visually confirming the successful preparation of CCP (Fig. S1).

### 2.2 In vitro cellular toxicity and uptake

To evaluate whether the as-prepared nanodrugs were cytotoxic, H9c2 cells were incubated with each treatment group, and cell viability was assessed using the CCK-8 assay (Fig. 1I). For the blank lipid nanoparticles (LP) group, cell viability remained above 95% across all investigated concentrations, revealing that the delivery vehicle exhibited negligible cytotoxicity toward H9c2 cells. For the CCP and CP formulations, cell viability was maintained above 80% at concentrations of 7 μg/mL and 14 μg/mL, respectively, suggesting a favorable biosafety profile at these dosages (Fig. S2). However, as the concentration increased further, a gradual decline in cell viability was observed, reflecting a typical dose-dependent inhibitory effect.

**Fig. 2:**
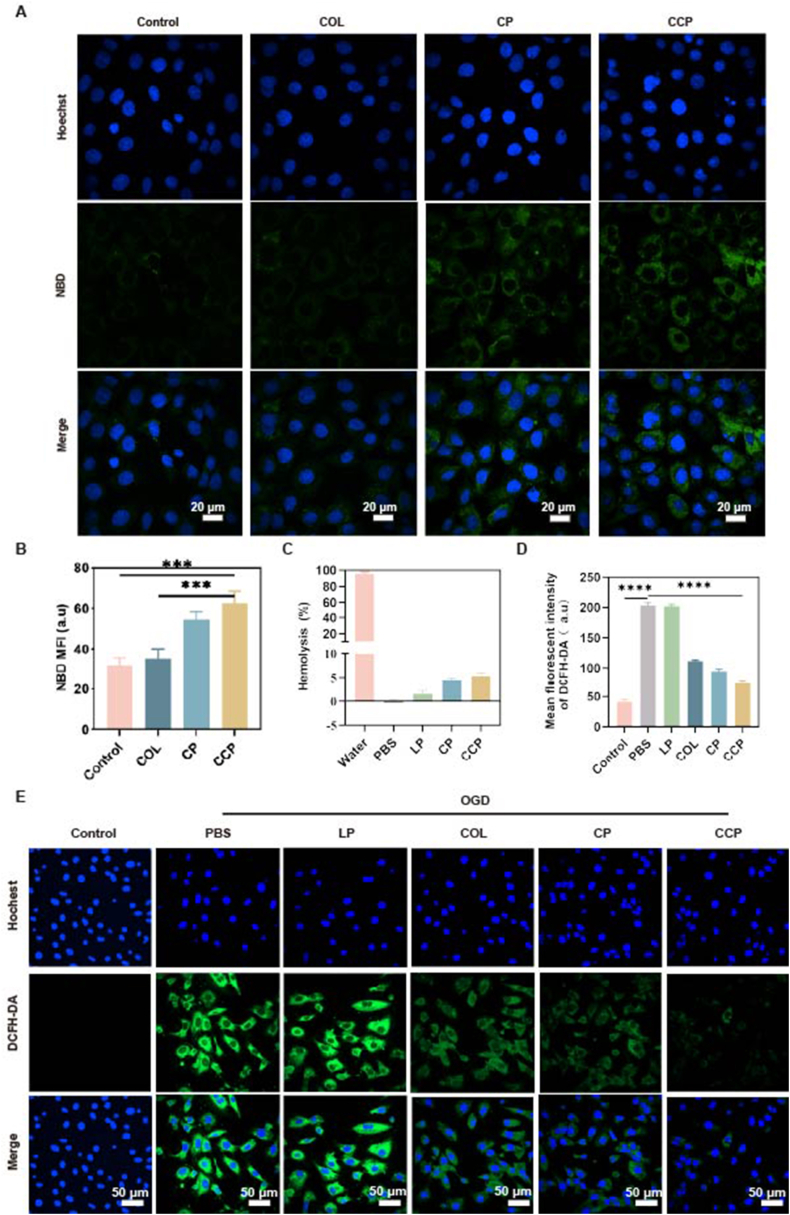
***In vitro* cellular uptake and detection of ROS level. (**A) CLSM analysis (scale bar: 20 μm). (B) Fluorescence quantitative analysis of Fig. 2A. (C) the hemolysis rate of each group. (D) Fluorescence quantitative analysis of Fig. 2E. The data results are presented as the mean standard deviation. (E) CLSM analysis (scale bar: 50 μm) (*n* = 3). Data are presented as mean ± SD (n = 3). Statistical significance was analyzed by one-way ANOVA with Dunnett's post hoc test. ∗∗∗∗*P* < 0.0001 vs. MI group.

To evaluate whether liposomal encapsulation and CHP-functionalization could augment internalization efficiency, H9c2 cells were incubated for 2, 4, and 8 h with 4-Chloro-7-nitro-2,1,3-benzoxadiazole (NBD)-labeled COL (COL-NBD), synthesized via a nucleophilic substitution reaction to facilitate high-resolution visualization. Confocal laser scanning microscopy (CLSM) revealed that the green fluorescence signal within the cytoplasm increased progressively with time, substantiating a time-dependent uptake behavior across all investigated groups (Fig. 2A). A close comparison further revealed that CCP-treated cells exhibited a markedly higher fluorescence intensity than the free COL and CP groups at each predetermined interval (Fig. 2B), providing definitive evidence that the CHP modification enhances myocardial-specific targeting and cytoplasmic internalization.

Furthermore, hemolysis assays confirmed the excellent hemocompatibility of the nanoplatforms (Fig. 2C). All investigated formulations (LP, CP, and CCP) exhibited minimal hemolytic activity, with values remaining well below the 5% safety limit, highlighting their suitability for systemic administration. This favorable biosafety profile, synergistic with the enhanced uptake capacity, underscores that these engineered nanomedicines are highly promising candidates for targeted myocardial repair in the context of *in vivo* heart injury.

Intracellular redox imbalance, typically triggered by an excessive burst of ROS, constitutes a critical determinant of cell death in the MI model established via oxygen-glucose deprivation (OGD) (Fig. S3A). To evaluate the oxidative status of H9c2 cells across various treatment cohorts, the DCFH-DA fluorescent probe was utilized, which is oxidized into green-fluorescent DCF in the presence of ROS. As visualized by CLSM, the PBS-treated OGD group manifested an intense green fluorescence signal, indicating severe ROS accumulation (Fig. 2E). In contrast, treatment with CP and, more significantly, CCP markedly quenched this fluorescence intensity, suggesting that these formulations effectively suppressed the OGD-induced oxidative surge. Quantitative analysis of the mean fluorescence intensity (MFI) substantiated that CCP treatment significantly reduced ROS production compared to the PBS and LP groups (Fig. 2D). Taken together, these results substantiate that the as-prepared nanomedicines, particularly the CCP, possess potent ROS-scavenging capabilities and can effectively counteract oxidative damage, thereby functioning as robust cytoprotective agents during myocardial injury.

### 2.3 In vivo cytotoxicity and intracellular uptake

**Fig. 3:**
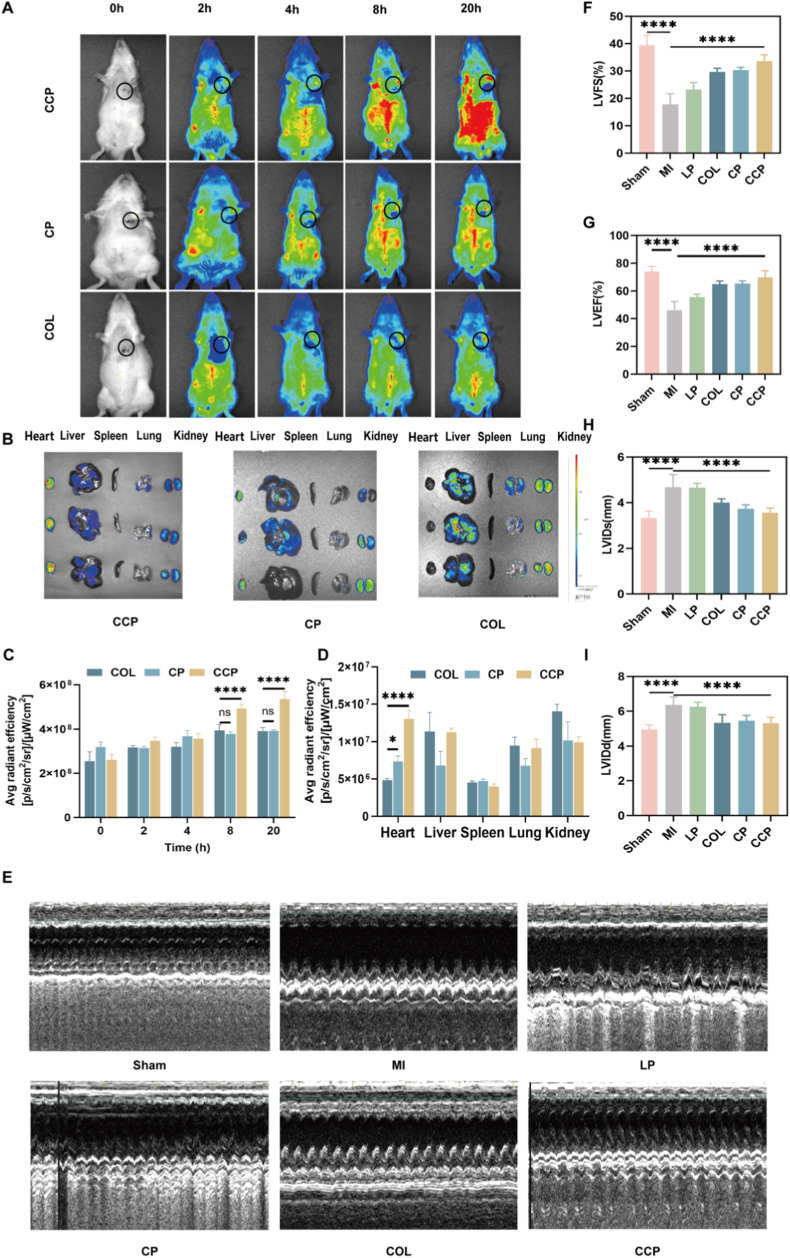
***In Vivo* Fluorescence Intensity and Evaluation of Cardiac Function.** (A) *in vivo* imaging results of rats at 0 h, 2 h, 4 h, 8 h, and 20 h. (B) *ex vivo* imaging results of rat organs. (C) the quantitative analysis of fluorescence intensity of Fig. 3A. (D) *in vivo* the quantitative analysis of fluorescence intensity in the heart, liver, spleen, lungs, and kidneys. Data results are presented as mean ± standard deviation (*n* = 3). (E) echocardiograms of the left atrium. (F-I) present statistical graphs of LVEF, LVFS, LVEF, LVIDs and LVIDd of Fig. 4A. Data are presented as mean ± SD (n = 3). Statistical analysis was performed using two-way ANOVA followed by post hoc multiple comparisons. ns, not significant; ∗*P* < 0.05; ∗∗∗∗P < 0.0001 vs. MI group.

The successful establishment of the MI model was rigorously validated by hallmark ST-segment elevation on ECG and immediate regional myocardial blanching following LAD ligation (Fig. S3B). To visually evaluate the *in vivo* targeting capacity and biodistribution of the nanomedicines, real-time NIR fluorescence imaging was conducted on MI rats at 0, 2, 4, 8, and 20 h post-injection (Fig. 3A). As illustrated in the whole-body images, fluorescent signals became clearly discernible 2 h after administration and reached maximum abundance at 20 h. Notably, a close comparison revealed that the CCP group manifested a significantly higher fluorescence intensity than the CP and COL groups at both 8 h and 20 h, accompanied by a prolonged signal duration in the cardiac region (*P* < 0.001; Fig. 3C). Such observations substantiated that the CCP formulation possessed superior *in vivo* distribution and heart-specific targeting capabilities (Fig. 3B). Subsequently, major organs and heart tissues were isolated at 20 h post-injection for ex vivo fluorescence imaging to further verify the site-specific accumulation. Compared with the liver, spleen, lungs, and kidneys, the heart in the CCP group exhibited a remarkably enhanced fluorescence signal (Fig. 3D), which was *ca.* Two-fold higher than that of the free COL group. Collectively, these results demonstrate that the CHP-modified nanoplatform facilitates efficient drug delivery across biological barriers and achieves selective enrichment within the ischemic myocardium.

#### 2.3.1 In vivo biosecurity

To evaluate the systemic biosafety of CCP *in vivo*, histopathological and biochemical analyses were performed. Histological examination via H&E staining revealed that liver and kidney sections maintained regular cellular architecture; the spleen showed distinct pulp demarcation, and the lungs exhibited intact alveolar structures (Fig. S4A). In line with these histological observations, hepatic and renal functional indicators—specifically ALT, AST, CREA, and UREA—remained within the normal physiological ranges for SD rats across all investigated cohorts (Fig. S4B–E). The absence of significant biochemical deviations or tissue damage underscores that the as-prepared nanomedicines do not compromise organ integrity, further substantiating their favorable biocompatibility for potential clinical translation.

### 2.4 CCP alleviates cardiac dysfunction and adverse remodeling Post-MI

To evaluate the therapeutic impact of CCP on post-MI cardiac performance, echocardiographic assessments were systematically performed at 28 days post-infarction (Fig. 3E). Key parameters, including LVEF, LVFS, LVEDD, and LVESD, were monitored to characterize heart structure and contractile function. As anticipated, the MI group manifested profound cardiac dysfunction, characterized by a substantial decline in LVEF (ca. 46.25%) compared with the Sham group (74.05%, *P* < 0.001), indicating successful establishment of the MI model. Intriguingly, both CP and CCP administrations significantly mitigated MI-induced deterioration. However, the CCP demonstrated a markedly superior protective effect. Specifically, CCP-treated rats manifested significantly elevated LVEF (69.88%) and LVFS (33.56%) compared with the untreated MI cohort (*P* < 0.001), demonstrating a marked recovery of myocardial contractile function. While COL and CP showed moderate improvements (LVEF of 65.02% and 65.31%, respectively), their efficacy was notably less pronounced than that of the CCP system (Fig. 3F–I). Collectively, these data substantiate that the CCP platform effectively preserves ventricular geometry and mitigates post-infarction cardiac dysfunction.

### 2.5 CCP attenuates pathological remodeling, fibrosis, and inflammatory cascades Post-MI

**Fig. 4:**
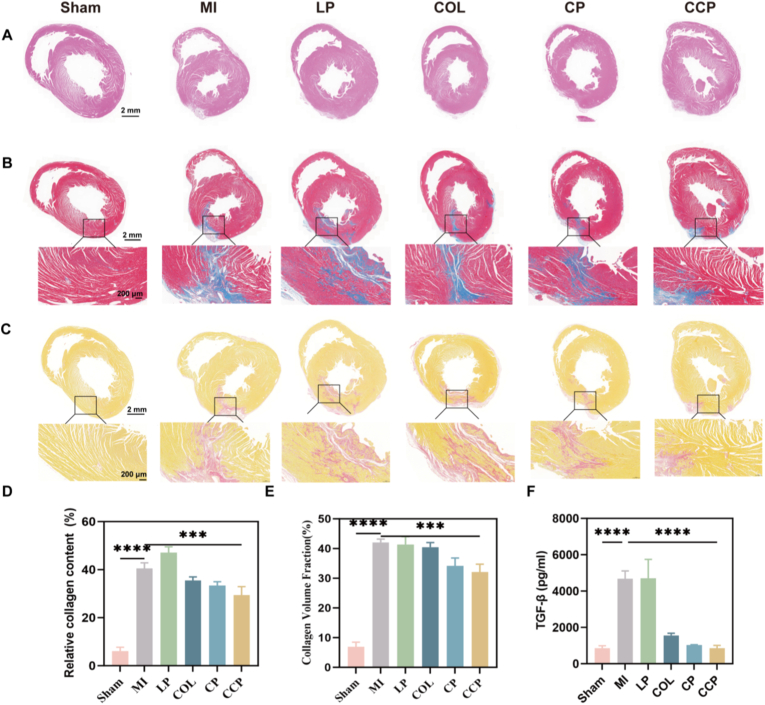
**The impact on fibrosis and inflammatory levels.** (A) the H&E staining (scale bar: 2 mm). (B) the Masson staining (scale bar: 200 μm). (C) the Sirius Red staining (scale bar: 200 μm) (A) the H&E staining (scale bar: 2 mm). (D) the quantitative analysis of fibrotic area. (E) the quantitative analysis of relative collagen content; (F) the serum levels of TGF-*β.* Data are presented as mean ± SD (n = 3). Multiple group comparisons were performed using one-way ANOVA followed by Dunnett's multiple comparisons test. ∗*p* < 0.05, ∗∗*p* < 0.01, ∗∗∗*p* < 0.001,∗∗∗∗*p* < 0.0001 vs. MI group. (For interpretation of the references to colour in this figure legend, the reader is referred to the Web version of this article.)

To comprehensively evaluate the therapeutic impact of CCP on post-infarction repair, we performed a multi-dimensional histological and biochemical analysis. First, H&E staining was employed to assess the overall myocardial architecture and ventricular geometry. As illustrated in the results, the MI and LP groups manifested severe myocardial fiber disarray and characteristic ventricular wall thinning. In contrast, CCP treatment significantly preserved the myocardial structural framework and attenuated the infiltration of inflammatory cells within the peri-infarct zone, suggesting a robust protective effect against acute tissue injury (Fig. 4A).

The preservation of myocardial integrity was further quantified by assessing extracellular matrix (ECM) remodeling using Masson's trichrome and Sirius Red staining (Fig. 4B and C). As anticipated, the Sham group exhibited negligible collagen deposition, whereas the MI cohort showed extensive collagen accumulation and dense interstitial fibrosis, reflecting a maladaptive fibrotic response. While COL and CP treatment partially reduced collagen density, the CCP platform emerged as the most effective intervention. Specifically, in the CCP group, collagen fibers were significantly attenuated and evenly distributed without massive scar-like aggregation (Fig. 4D and E). These morphological improvements substantiate that CCP effectively intercepts the transition from acute necrosis to chronic pathological fibrosis, thereby maintaining the biomechanical stability of the infarcted heart.

These histopathological findings were further corroborated by the systemic and local modulation of the inflammatory microenvironment. Compared with the Sham group, the MI and LP cohorts showed significant elevations in key molecular drivers of maladaptive remodeling, including TGF-*β,* IL*-6,* and TNF-*α* (*P* < 0.001). Notably, CCP treatment demonstrated the most potent inhibitory effect, manifesting a markedly superior capacity to suppress the expression of these pro-inflammatory cytokines compared to both the COL and CP controls (*P* < 0.001; Fig. 4F and Fig. S5). Collectively, these results reveal that the CCP achieves superior cardioprotection by concurrently quenching the inflammatory cascade and blocking TGF-*β*-mediated fibrotic programs, which ultimately orchestrates a transition from a pathological niche to a restorative cardiac environment.

### 2.6 CCP mitigates maladaptive myocardial sympathetic nerve remodeling

**Fig. 5:**
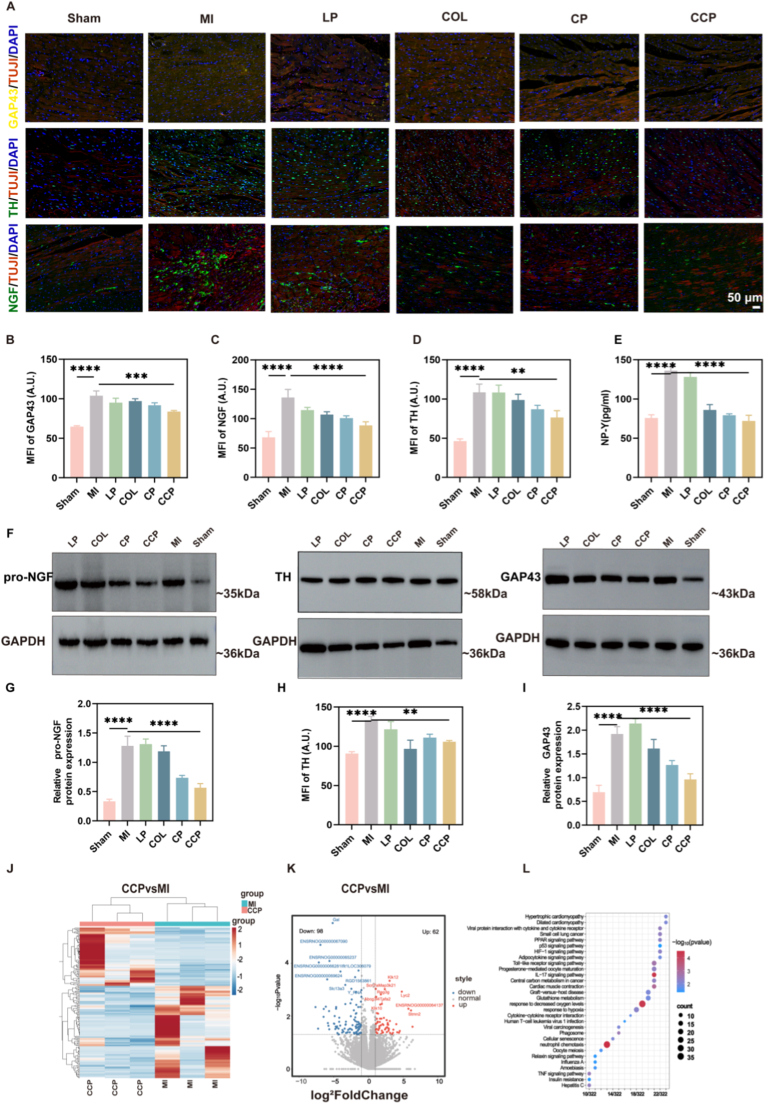
**Neural Structural Dynamics and Remodeling in CCP. (A)** Immunofluorescence analysis of myocardial tissue for GAP43, NGF, and TH (scale bar: 100 μm). (B-D) the quantitative analysis of the average fluorescence intensity of GAP43, NGF, and TH. (E) the serum NP-Y levels. (F) Representative Western blot images of neural remodeling markers. (G) Densitometric quantification of relative pro-NGF protein expression. (H) Densitometric quantification of relative TH protein expression. (I) Densitometric quantification of relative GAP43 protein expression. Transcriptomic analysis of CCP and MI (*n* = 3) group Gene Expression Changes in CCP and MI Groups: (J) Heatmap, (K) Volcano Plot, and (L) Enrichment Analysis. ∗∗∗∗*P* < 0.0001. Data are presented as mean ± SD (n = 3). Multiple group comparisons were performed using one-way ANOVA followed by Dunnett's multiple comparisons test. ∗*p* < 0.05, ∗∗*p* < 0.01, ∗∗∗*p* < 0.001, ∗∗∗∗*p* < 0.0001 vs. MI group.

To investigate the impact of the targeted nanoplatform on post-MI neural remodeling, immunofluorescence staining and Western blot analysis were performed to evaluate key markers of sympathetic nerve activity and neuroplasticity. Specifically, we monitored Tyrosine Hydroxylase (TH), a rate-limiting enzyme for catecholamine biosynthesis, along with Growth-Associated Protein 43 (GAP43) and Nerve Growth Factor (NGF), which collectively orchestrate pathological nerve sprouting and survival (Fig. 5A). The results underscore that the MI group manifested a pronounced sympathetic surge and maladaptive neuroplasticity, characterized by significant increases in the fluorescence intensities of GAP43, NGF, and TH compared with the Sham group (Fig. 5B–D). Furthermore, the levels of Neuropeptide Y (NPY)—a sympathetic co-transmitter linked to vascular tone and cardiac stress—exhibited a similar downward trend following CCP administration (Fig. 5E). This aberrant neural remodeling is a critical driver of lethal arrhythmias and adverse cardiac outcomes. While COL and LP groups showed persistent elevation of these markers, treatment with CCP effectively suppressed the MI-induced neuroplastic surge. Notably, CCP treatment resulted in a substantial reduction of these neural markers compared to the MI group, demonstrating a superior capacity to stabilize the cardiac autonomic microenvironment relative to CP.

The spatial reduction in neural markers observed via IF was further substantiated by the quantitative assessment of protein expression. Consistent with the imaging data, Western blot analysis revealed a significant accumulation of pro-NGF (ca. 35 kDa) in the MI cohort, which was markedly attenuated by CCP treatment (Fig. 5F–I). Collectively, these findings demonstrate that CCP effectively intercepts the inflammatory-neural signaling axis, thereby mitigating pathological sympathetic hyperinnervation and providing a neuro-protective environment essential for functional myocardial repair.

### 2.7 Transcriptomic profiling reveals the molecular underpinnings of CCP-mediated repair

To gain a comprehensive understanding of the molecular mechanisms underlying CCP-mediated cardioprotection, whole-transcriptome RNA sequencing was performed on cardiac tissues from the MI and CCP cohorts (Fig. 5J). Differential expression analysis identified a distinct transcriptomic shift, with 62 upregulated and 98 downregulated genes in the CCP group relative to the MI group. As illustrated in the volcano plot (Fig. 5K), the majority of these differentially expressed genes (DEGs) were significantly downregulated, revealing that the CCP intervention primarily exerts its therapeutic effects by intercepting pathological signaling cascades associated with post-MI injury, particularly those governing inflammatory and fibrotic responses.

To further interpret the biological implications of these transcriptomic alterations, KEGG pathway enrichment analysis was conducted (Fig. 5L). Notably, the DEGs were predominantly enriched in pathways intimately linked to adverse cardiac remodeling and immune activation, specifically Hypertrophic Cardiomyopathy and Viral Protein Interaction with Cytokines and Cytokine Receptors. The significant modulation of the hypertrophic cardiomyopathy pathway substantiates the efficacy of CCP in attenuating maladaptive myocyte growth, a structural hallmark of heart failure. Simultaneously, the enrichment of cytokine-receptor interaction pathways provides robust molecular evidence for the systemic and local suppression of immune-mediated inflammatory signaling, which aligns with our observed reductions in pro-inflammatory mediators and neuroplastic markers.

Collectively, these transcriptomic insights elucidate the multi-dimensional protective effects of the heart-targeting CCP platform. The coordinated downregulation of genes involved in cardiac hypertrophy and cytokine signaling suggests that CCP does not merely act as a passive delivery vehicle but rather orchestrates a systemic transition of the ischemic myocardium from a pro-inflammatory, maladaptive state toward a stabilized, restorative environment. This molecular evidence further validates the potential of CCP as a high-precision therapeutic for functional myocardial recovery.

## 3 Discussion

This study introduces a high-precision therapeutic strategy for MI utilizing CCP. Our findings demonstrate that CCP significantly augments targeted drug delivery to the ischemic myocardium, thereby improving contractile performance, attenuating fibrosis, and uniquely inhibiting maladaptive sympathetic nerve remodeling [23,24]. By overcoming the hurdles of inefficient distribution and systemic toxicity associated with free COL, CCP establishes a localized, high-efficiency platform for myocardial repair [25,26].

In terms of delivery technology, our results extend the current paradigm of LNPs. Unlike conventional LNPs that often suffer from hepatic sequestration and limited cardiac accumulation, the incorporation of CHP introduces superior specificity [27]. By binding to endothelial markers exposed during ischemia, CCP bypasses the reticuloendothelial system to achieve selective myocardial enrichment, optimizing the therapeutic index of COL [28].

Beyond delivery efficiency, transcriptomic profiling provides profound insights into how CCP reconfigures the post-MI microenvironment. Traditionally, the advantage of COL is attributed to broad anti-inflammatory effects [17,29]. However, our data reveals a more nuanced, upstream molecular logic. Intriguingly, cardioprotection appears driven by a subset of unconventional genes rather than canonical fibrotic signaling. Specifically, the downregulation of early triggers (Map3k21, Klk10/12) and recruitment markers (Lyz2, Reg3g) suggests that CCP intercepts the pathological cascade at its initiation. Most significantly, the modulation of Stmn2, Tafa2, and Scn7a underscores a sophisticated regulation of neuro-immune interaction and ionic homeostasis—dimensions frequently overlooked in conventional studies. While Stmn2 and Tafa2 orchestrate neuronal plasticity, Scn7a is essential for maintaining the ionic balance required for rhythmic stability [30,31].

This holistic reconfiguration provides a robust explanation for our pathway enrichment results. The prevalence of hypertrophic and dilated cardiomyopathy pathways over narrow fibrotic terms suggests that CCP orchestrates a systemic transition from a maladaptive state toward a restorative landscape. This shift is particularly evident in the inhibition of sympathetic hyperinnervation and the preservation of cardiac geometry.

Despite these promising results, several limitations warrant consideration [32] While the therapeutic efficacy in rat models is evident, clinical validation is required to confirm the long-term safety and pharmacokinetics of the CCP platform in humans [33,34]. Additionally, while we observe protein-level changes (e.g., NGF and TH) we acknowledge that the lack of direct mRNA-level validation (qPCR) for the identified differentially expressed genes remains a limitation of this study. Future investigations incorporating multi-tier genetic validation will be beneficial to further confirm the precise transcriptional regulatory networks of CCP. Furthermore, although we have identified key upstream genetic regulators, the complete interactome governing the transition from a pathological to a restorative cardiac landscape remains to be fully decoded potentially through future spatial transcriptomics or single-cell analysis. Addressing these gaps will be essential for advancing the clinical translation of targeted nanotherapy in cardiovascular medicine [35].

## 4 Conclusion

In summary, this study substantiates that the incorporation of CHP into COL-loaded LNPs significantly enhances myocardial targeting and therapeutic efficacy post-MI. By concurrently quenching oxidative stress and modulating a distinct transcriptomic signature of 98 genes involved in innate inflammation and neuroplasticity, the CCP platform successfully reconfigures the pathological cardiac niche into a restorative environment. These findings not only demonstrate the superiority of targeted delivery over conventional COL treatments but also introduce a transformative approach that integrates biomimetic engineering with multi-dimensional molecular intervention. Collectively, this research provides a robust foundation for the development of high-precision nanomedicines to prevent maladaptive remodeling and improve clinical outcomes in patients with ischemic heart disease.

## 5 Experimental section

### 5.1 Materials

CHL (CAS 57-88-5), SPC (CAS 8002-43-5), and COL (CAS 64-86-8) were purchased from Shanghai Yuanye Bio-Technology Co., Ltd. (Shanghai, China). The cardiac-homing peptide conjugate DSPE-PEG2000-CSTSMLKAC was obtained from Shanghai Qiangyao Biotechnology Co., Ltd. (Shanghai, China). Analytical-grade methanol (CAS 67-56-1), dichloromethane (CAS 75-09-2), and *n*-hexane (CAS 110-54-3) were supplied by Shanghai Aladdin Biochemical Technology Co., Ltd. (Shanghai, China). NBD-Cl (CAS 10199-89-0) was purchased from Shanghai Macklin Biochemical Technology Co., Ltd. (Shanghai, China) for the synthesis of fluorescently labeled colchicine (COL-NBD). Hoechst 33342 (CAS 23491-52-3), phosphate-buffered saline (PBS, 1 × ), and dimethyl sulfoxide (DMSO, cell-culture grade, CAS 67-68-5) were obtained from Beijing Solarbio Science & Technology Co., Ltd. (Beijing, China). High-glucose Dulbecco's Modified Eagle Medium (DMEM), penicillin/streptomycin solution (100 KU/L), and 0.25% trypsin were purchased from Biological Industries (BI) Bio-Technology Co., Ltd. (Israel). Fetal bovine serum (FBS) was supplied by Gibco (Thermo Fisher Scientific, Waltham, MA, USA). Cell Counting Kit-8 (CCK-8) was purchased from Wuhan Boster Biological Technology Co., Ltd. (Wuhan, China).

Masson's trichrome staining kit and Sirius Red staining kit were purchased from Fuzhou Maixin Biotechnology Development Co., Ltd. (Fuzhou, China) and Beijing Solarbio Science & Technology Co., Ltd. (Beijing, China), respectively. Primary antibodies for Western blotting—rabbit anti-TH (tyrosine hydroxylase), rabbit anti-GAP43 (growth-associated protein 43), and rabbit anti-pro-NGF (pro-nerve growth factor)—together with HRP-conjugated goat anti-rabbit secondary antibody were purchased from Abcam (Cambridge, UK) and used according to the manufacturer's instructions. Anesthetics included Zoletil® (Virbac S.A., Carros, France) and Suminxin (compound xylazine hydrochloride, Dunhua Shengda Animal Pharmaceutical Co., Ltd., China). 75% ethanol was obtained from Henan Huakai Biotechnology Co., Ltd. (Henan, China).

H9c2 rat cardiomyoblast cells were maintained from laboratory cryopreserved stocks. Male Sprague-Dawley (SD) rats (200 ± 10 g) were supplied by the Experimental Animal Center of Xinjiang Medical University (Urumqi, China). Cell-culture consumables (dishes, flasks, and multi-well plates) were purchased from Wuxi NEST Biotechnology Co., Ltd. (Wuxi, China). All other reagents and solvents were of analytical grade and used without further purification.

### 5.2 Instrument

The particle sizes and zeta potentials of CP and CCP were measured using a laser particle size and zeta potential analyzer (Zetasizer Nano ZS90, Malvern, UK). The morphology of CP and CCP was observed by transmission electron microscopy (TEM, JEM-2100F, JEOL, Japan). Fourier transform infrared spectroscopy (FTIR, VERTEX 70, Bruker, Germany) and X-ray diffraction (XRD, D8 Advance, Bruker, Germany) were employed to obtain the corresponding spectra. Fluorescence stability was assessed using a fluorescence spectrophotometer (F97Pro, Luster Light, China). The ultraviolet absorption and fluorescence values at specific wavelengths were measured with a multifunctional microplate reader (SpectraMax iD5, Molecular Devices, USA). Finally, confocal laser scanning microscopy (CLSM, A1R HD25, Nikon, Japan) was used to acquire images, and *in vivo* fluorescence distribution was evaluated with a small-animal imaging system (IVIS Lumina XRM5, PerkinElmer, USA).

### 5.3 Construction of CP and CCP

Soybean lecithin (7 mg), cholesterol (1 mg), and COL (1 mg) were dissolved in a methanol/dichloromethane mixture (2:1, v/v) to a final volume of 12 mL using ultrasonication for 3 min. The solution was evaporated in a 50 °C oil bath to form a thin film, which was then redissolved in n-hexane to obtain a homogeneous 10 mL mixed solution. The resulting solution was sequentially filtered through 0.80, 0.45, and 0.22 μm membranes to obtain the CP suspension. After re-evaporation and hydration with 500 mL distilled water, the product was sonicated at 20 kHz for 10 min to yield a clear solution, which was then stored at 4 °C. For CCP preparation, the CP suspension was mixed with 0.45 mg DSPE-PEG2000-CSTSMLKAC and stirred at 500 rpm for 6 h at room temperature (25 ± 2 °C).

### 5.4 In vitro fluorescence stability

In vitro fluorescence stability of CP and CCP was evaluated using fluorescence spectroscopy, with the same concentration of NBD serving as the control group.

### 5.5 Culture and maintenance of H9c2 rat cardiomyoblast cells

H9c2 cardiomyoblasts were cryopreserved in liquid nitrogen within the laboratory and routinely recovered for experiments. Cells were cultured in high-glucose Dulbecco's Modified Eagle Medium (DMEM) supplemented with 10% (v/v) fetal bovine serum (FBS) and 1% penicillin–streptomycin, and maintained at 37 °C in a humidified atmosphere containing 5% CO_2_. Cells were passaged at 70–80% confluence using 0.05% trypsin and routinely subcultured at a split ratio of 1:3 to 1:10 depending on the growth rate.

### 5.6 In vivo targeting effect

To study the targeting effect of CCP, CP, and COL, these substances were injected into Sprague-Dawley rats for NIR fluorescence imaging studies. Drug accumulation was monitored at predetermined time points using an *in vivo* imaging system. Subsequently, the primary tissue samples were harvested for ex vivo fluorescence imaging.

### 5.7 Establishment of the MI model and in vivo evaluation

Male Sprague-Dawley rats (200 ± 10 g) were randomly assigned to six cohorts: Sham, MI, LP, COL, CP, and CCP. All animals were acclimatized for 7 days and fasted for 12 h with ad libitum access to water prior to surgery. On the day of the procedure, rats were anesthetized via intramuscular injection of a freshly prepared Zoletil®-based combined anesthetic (30–50 mg/kg), which consistently maintained a surgical plane of anesthesia. Following endotracheal intubation, mechanical ventilation was initiated (tidal volume: 10 mL/kg; respiratory rate: 50 breaths/min; I: E ratio: 1:2). A left thoracotomy was then performed at the third to fourth intercostal space. Myocardial infarction was induced by permanent ligation of the left anterior descending (LAD) coronary artery using a 6-0 silk suture over a 5-mm latex tube. The success of the model was confirmed by immediate regional myocardial blanching and characteristic ST-segment elevation on postoperative electrocardiography (ECG).

Postoperatively, rats were closely monitored in a temperature-controlled recovery environment until full ambulation was restored. No additional analgesics were administered, as the duration of the initial anesthesia provided sufficient coverage during the early recovery phase, thereby minimizing potential pharmacological interference with inflammatory or neural signaling outcomes. Crucially, to ensure the full establishment of the pathological inflammatory microenvironment and to assess target-specific homing, the liposomal formulations (200 μg/kg) or an equivalent volume of saline were administered intravenously via the tail vein at 48 h post-infarction. All formulations were administered intravenously via tail vein injection at a dose of 200 μg/kg once daily.

### 5.8 Transcriptome sequencing and bioinformatics analysis

Myocardial tissues from MI and CCP rats (n = 3 per group) were sent for RNA sequencing (HaploX Biotechnology, Jiangxi, China). RNA purity, concentration, and integrity were assessed using NanoDrop, Qubit, and Agilent TapeStation, respectively. Poly(A) + mRNA was enriched using Oligo(dT) beads, reverse-transcribed, converted into double-stranded cDNA, end-repaired, adapter-ligated, PCR-amplified, and size-selected (∼200 bp). Libraries were quantified by KAPA qPCR, fragment size assessed with TapeStation, and qualified libraries sequenced. Clean reads were quality-controlled and mapped to the reference genome for downstream analysis.

### 5.9 Statistical analysis

The hydrodynamic diameter of nanoparticles was measured by dynamic light scattering (DLS), which reflects the particle core together with the surface coating and solvation layer in aqueous solution.

Data were analyzed using GraphPad Prism (Version 8.0), R software (Version 4.3.3), and expressed as the mean ± SD of at least three repeated measurements. Two-sided t-tests were performed to analyze the data. A *p*-value <0.05 was considered statistically significant. Statistical significance was indicated as follows: ∗p < 0.05, ∗∗*p* < 0.01, ∗∗∗*p* < 0.001, and ∗∗∗∗*p* < 0.0001.

## Ethics approval and consent to participate

The animal study protocol was reviewed and approved by the Institutional Animal Care and Use Committee (IACUC) of 10.13039/501100004880Xinjiang Medical University (No. IACUC-JT-20250911-01). All experimental procedures adhered to the Guide for the Care and Use of Laboratory Animals published by the US 10.13039/100000002National Institutes of Health (NIH Publication No. 85-23, revised 1996) and the ARRIVE guidelines.

## CRediT authorship contribution statement

**Xing Zhang:** Conceptualization, Data curation, Writing – original draft. **Wenhua Xu:** Data curation. **Jie Song:** Software. **Huanhuan Ding:** Data curation, Software. **Shujie Yu:** Software. **Nan Yang:** Data curation. **Dilare Taiwaikuli:** Formal analysis. **Yemin Chen:** Software. **Yanmei Lu:** Investigation, Resources, Supervision. **Baopeng Tang:** Resources, Supervision. **Zhongxiong Fan:** Conceptualization, Data curation, Funding acquisition, Supervision. **Xianhui Zhou:** Conceptualization, Data curation, Funding acquisition, Supervision.

## Declaration of competing interest

The authors declare that they have no known competing financial interests or personal relationships that could have appeared to influence the work reported in this paper.

## Data Availability

The authors do not have permission to share data.
