# Supplementary material for: Cardiac-homing peptide-targeted colchicine-based drugstores with inhibiting inflammatory response for myocardial infarction improvement
Source: Mater Today Bio. 2026 May 13;38:103212. doi: 10.1016/j.mtbio.2026.103212 (PMC13214554; doi:10.1016/j.mtbio.2026.103212)
Supplement: Multimedia component 1 [file mmc1.docx]

***Supplementary Material***

**Cardiac-homing peptide-targeted colchicine-based drugstores with inhibiting inflammatory response for myocardial infarction improvement**

*Xing Zhang^1,#^, Wenhua Xu^1,4,#^, Jie Song^1,#^, Huanhuan Ding^1^, Shujie Yu^2,3^, Nan Yang^2,3^, Dilare Taiwaikuli^1^, Yemin Chen^1^, Yanmei Lu^1^, Baopeng Tang^1,^*, Zhongxiong Fan^2,^*, Xianhui Zhou^1,5^**

^1^ Department of Cardiac Pacing and Electrophysiology & Xinjiang Key Laboratory of Cardiac Electrophysiology and Remodeling, The First Affiliated Hospital of Xinjiang Medical University, Urumqi, 830054, China

^2^ School of Pharmaceutical Sciences, Institute of Materia Medica, Xinjiang University, Urumqi 830017, China

^3^ Xinjiang Key Laboratory of Biological Resources and Genetic Engineering, College of Life Science and Technology, Xinjiang University, Urumqi 830017, China

^4^ Department of Cardiology, Changji Prefecture People's Hospital in Xinjiang Uygur Autonomous Region, No.303 Yan'an Road, Changji City 831100, Xinjiang, China

^5^ School of Public Health, Xinjiang Medical University

***Corresponding authors**: Dr. Baopeng Tang, E-mail: tangbaopeng1111@163.com; Dr. Zhongxiong Fan, E-mail: [fanzhongxiong@xju.edu.cn](mailto:fanzhongxiong@xju.edu.cn); Dr. Xianhui Zhou, E-mail: zhouxhuiyf@163.com.

***Supplementary data***

***
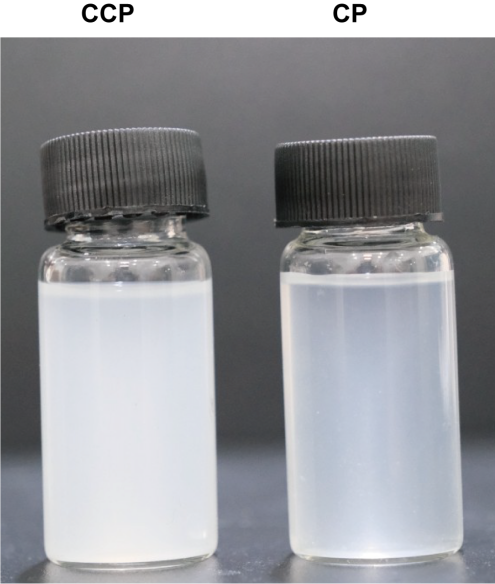
***

**Figure S1.** Photograph of **CCP** (left) **and CP (right)** solution

***
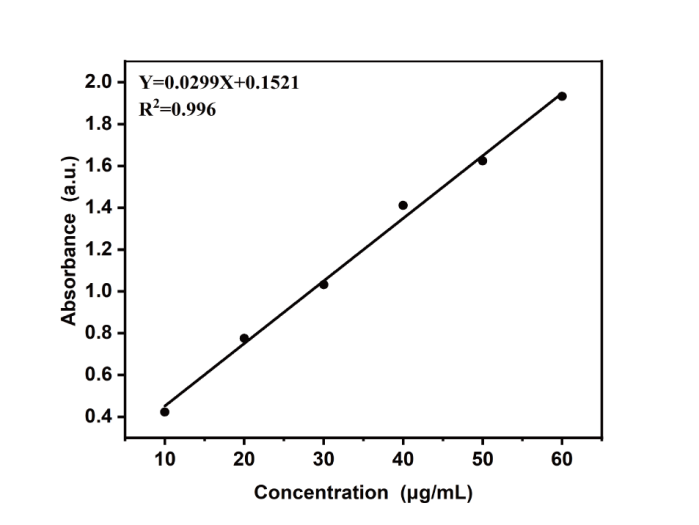
***

**Figure S2.** the standard curve for COL determination.

***
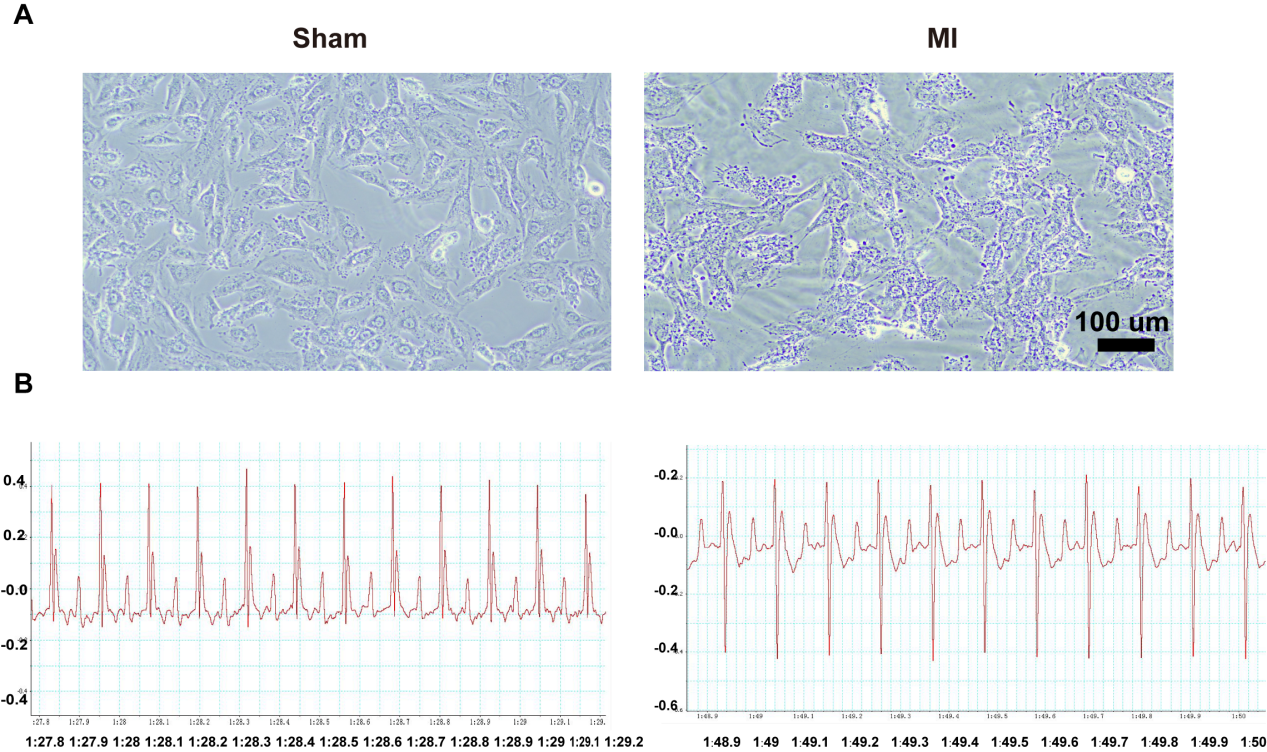
***

**Figure S3 (A)** Light Microscopy Images of Sham and OGD. (B) Electrocardiogram Characteristics of Sham and MI Groups.


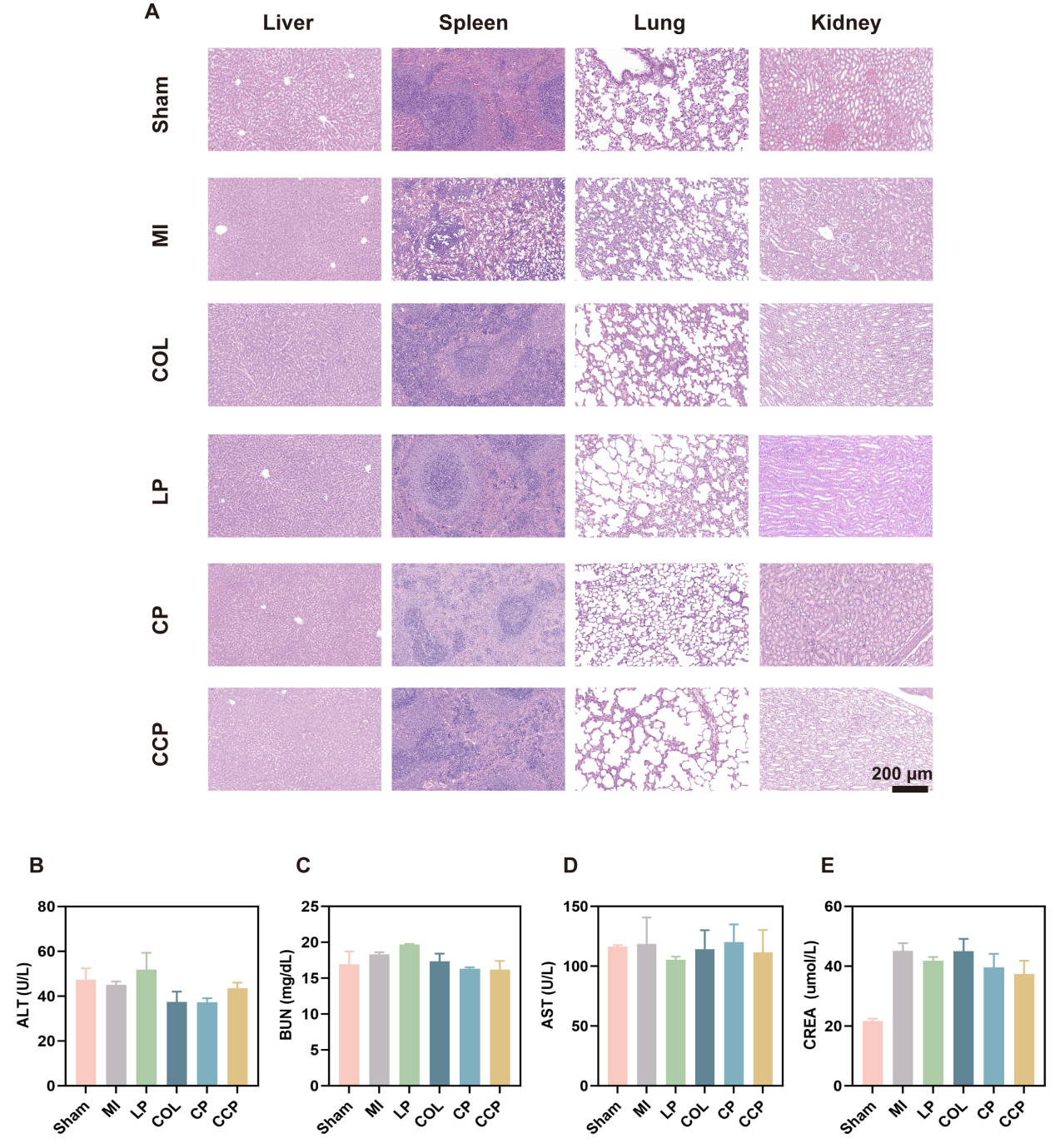


**Figure S4 (**A) H&E staining of the liver, spleen, lungs, and kidneys in each group (scale bar: 200 *μ*m). (B-E) comparative analysis of liver and kidney function, including ALT, BUN, AST, and CREA, across the groups. The data results are presented as the mean ± standard deviation (*n* = 3).


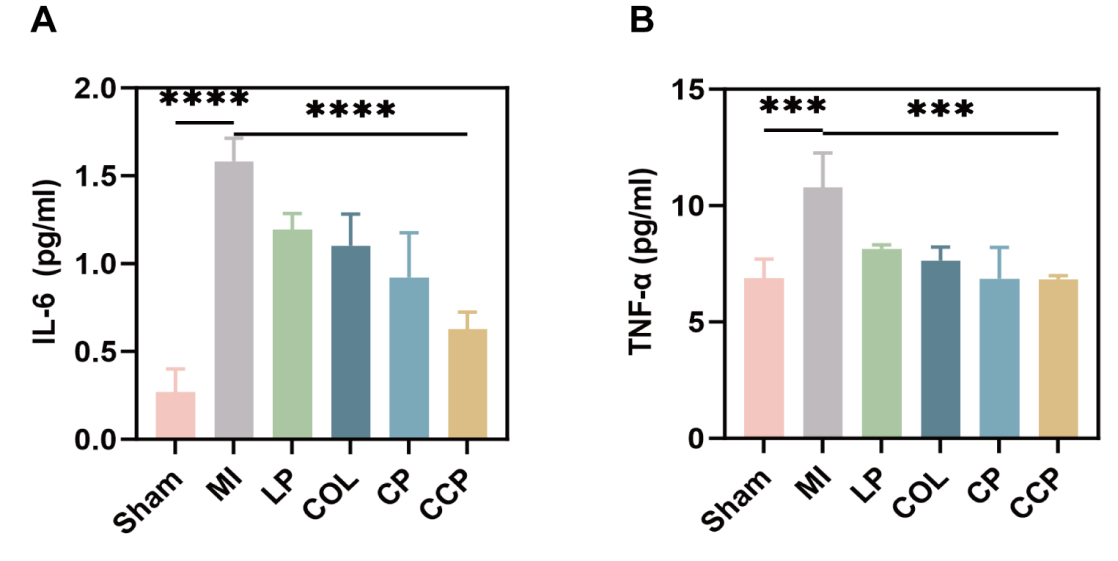


**Figure S5 (A)** the serum levels of IL-6.(B) the serum levels of TNF-α.The data results are presented as the mean ± standard deviation (*n* = 3). Data are presented as mean ± SD (n = 3).Statistical significance was analyzed by one-way ANOVA with Dunnett’s post hoc test.****p* < 0.001 ,*****p* < 0.0001 vs. MI group.
